# Using Population Genetic Theory and DNA Sequences for Species Detection and Identification in Asexual Organisms

**DOI:** 10.1371/journal.pone.0010609

**Published:** 2010-05-13

**Authors:** C. William Birky, Joshua Adams, Marlea Gemmel, Julia Perry

**Affiliations:** 1 Department of Ecology and Evolutionary Biology, Biological Sciences West, The University of Arizona, Tucson, Arizona, United States of America; 2 Graduate Interdisciplinary Program in Genetics, Biological Sciences West, The University of Arizona, Tucson, Arizona, United States of America; McGill University, Canada

## Abstract

**Background:**

It is widely agreed that species are fundamental units of biology, but there is little agreement on a definition of species or on an operational criterion for delimiting species that is applicable to all organisms.

**Methodology/Principal Findings:**

We focus on asexual eukaryotes as the simplest case for investigating species and speciation. We describe a model of speciation in asexual organisms based on basic principles of population and evolutionary genetics. The resulting species are independently evolving populations as described by the evolutionary species concept or the general lineage species concept. Based on this model, we describe a procedure for using gene sequences from small samples of individuals to assign them to the same or different species. Using this method of species delimitation, we demonstrate the existence of species as independent evolutionary units in seven groups of invertebrates, fungi, and protists that reproduce asexually most or all of the time.

**Conclusions/Significance:**

This wide evolutionary sampling establishes the general existence of species and speciation in asexual organisms. The method is well suited for measuring species diversity when phenotypic data are insufficient to distinguish species, or are not available, as in DNA barcoding and environmental sequencing. We argue that it is also widely applicable to sexual organisms.

## Introduction

In *The Origin of Species*, Charles Darwin showed how a single species can give rise to two different varieties and eventually to species by the accumulation of gradual changes. However, precisely because the changes are mainly gradual, Darwin's work raised a problem that has vexed biologists ever since: how can gradual changes in two lineages produce the apparently discrete morphological clusters that we call species? Darwin wrote: “Finally, varieties cannot be distinguished from species—except, first, by the discovery of intermediate linking forms; and, secondly, by a certain indefinite amount of difference between them; … but the amount of difference considered necessary to give to any two forms the rank of species cannot be defined” [1]. The problem described by Darwin led him, and some others since him, to believe that species are arbitrary divisions not clearly distinguishable from varieties. And yet it is widely recognized that organisms do tend to fall into fairly discrete clusters of individuals with similar phenotypes that are usually called species; they are often distinguished not only by their phenotypes but also by DNA sequences that also fall into discrete clusters. Moreover, sexual organisms exchange genes readily within clusters but not between them. These facts are widely accepted and biologists tend to treat species as fundamental units of biology; nevertheless defining species continues to be one of the most difficult and contentious problem in biology [2], [3], [4], [5]. Given the renewed interest and urgency in understanding and measuring the diversity of life and the impact of climate change on diversity, few biological problems are more important. In an earlier paper we presented a solution to Darwin's problem for asexual organisms and used it to define species in the bdelloid rotifers [6]. Here we update our model of speciation and the associated species criterion (the 4× rule) for delimiting species in asexual and clonal organisms [6]; see also [7]. Further, we apply the 4× rule to a diverse collection of asexual eukaryotes, showing that the formation of evolutionary species is a general property of asexual as well as sexual eukaryotes.

DNA sequences are increasingly being used in systematics and surveys of biological diversity, both to find clusters that can be called species (species detection) and to assign new specimens to previously identified species [8]. Sequences are even used to estimate the number of species or of unspecified units of biological diversity without knowledge of the organisms from which they came (environmental sequencing; e.g. [9]). Sequence data are applicable to any developmental stage or part of an individual, are not confounded by developmental polymorphism or phenotypic plasticity, and are less likely to be confounded by convergent evolution. Moreover they can be used when phenotypic traits are difficult or impossible to detect, or are simply unavailable as in environmental sequencing. Of course DNA sequences by themselves provide limited insight into the biology of organisms, and their use in systematics does not relieve the need to make detailed studies of the organisms whenever possible.

However, problems can arise when DNA sequences, phenotypes, or any other data are used for species detection without using a well-defined species concept or model of what species are. Assigning DNA sequences (and the individuals from which they are taken) to species will be an arbitrary and biologically meaningless exercise unless it is done with an operational species criterion corresponding to a rigorous theoretical model. The species criterion should take into account both the species concept and also the fact that species detection is based on small samples of individuals. If sampling statistics are not considered, one risks describing as species what are really subpopulations within species, or groups of closely related species. Moreover, comparative evolutionary studies of species are only useful when the species are comparable evolutionary units; this requires that they all be defined using the same species concept and criterion.

Here we focus on species in asexual and clonal organisms, partly because it is necessary to understand asexual speciation in order to study the evolutionary advantages of sex, and partly in the hope of finding clues to the nature of species in general and how to define them in sexuals by studying the simpler case of asexuals first. The absence of sexual reproduction with outcrossing makes it easier to define species using genotypes because every gene in an individual has the same evolutionary history, with rare exceptions; in contrast to sexual organisms, any gene can be used to group organisms into species and assign new specimens to those species. (Exceptions are genes showing the Meselson effect [10], [11] or paralogy which can result in phylogenetic trees that do not reflect the history of the organisms.) Theory shows that asexual organisms can form significant clusters or clades due to adaptation to different niches or to physical separation, the same factors that lead to speciation in sexual organisms [6], [12]. These clades are independently evolving populations or metapopulations as described by the original evolutionary species concept of Simpson [13]; they are also segments of independently evolving metapopulation lineages as described by the general lineage species concept [5], [14], [15], [16]. Note however that some later versions of the evolutionary species concept included the requirement that the species will continue to evolve independently in the future [17], which we can rarely know with any certainty. Also we do not accept some of the consequences of the general lineage species concept discussed by de Queiroz [16], for example that species can be contained within species.

**Figure 1:**
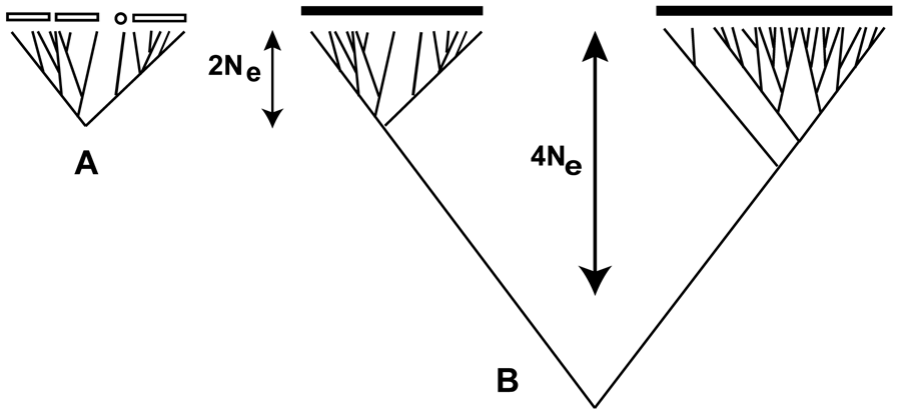
Speciation as seen in phylogenetic trees of asexual organisms descended from a single asexual founder. (*A*) A single species is an inclusive population that is limited to some number of individuals *N* by the carrying capacity of the environment and will show extinction of lineages due to stochastic processes. The effective population size *N_e_* is a smaller number reflecting the fact that different individuals are producing different numbers of offspring. This results in clades (open bars) and singlets (circle) separated by transient gaps with an average depth of 2*N_e_* generations. (*B*) A tree in which two lineages have been physically separated, e.g. by distance, or have undergone divergent selection for adaptation to different niches (or both) long enough to complete lineage sorting and become reciprocally monophyletic. The populations form two clusters separated by a gap that becomes deeper over time until it is much deeper than 2*N_e_* generations.

Our speciation model is summarized in Figure 1. Stochastic extinction of lineages within a single species (1A), which is equivalent to random genetic drift, will produce clades and singlets separated by gaps. Similar gaps and clusters can also be formed if the species is a metapopulation divided into demes connected by infrequent migration. However, all of the individuals in a species will be descended from a common ancestor on average 2*N*_e_ generations ago, where *N*_e_ is the effective population size. (Note that N_e_ can be defined to include not only random drift but also other factors affecting diversity, notably selection and population subdivision with migration and periodic extinction and recolonization of subpopulations [18]). Consequently the gaps formed by drift or population structure will be transient, with an average depth less than 2*N*_e_ generations. Because these gaps are transient, the population still forms a single arena for the forces of evolutionary genetics (random drift, mutation, and selection). Speciation (1B) begins when a species is split into two populations that are physically completely separated, e.g. by distance, or have undergone divergent selection for adaptation to different niches, or both. The populations will then form two clusters separated by a gap that deepens over time until it is much deeper than 2*N*_e_ generations. We consider that speciation is complete when the populations are separated by gaps too deep to be produced by random drift alone. If the two clusters are adapted to different niches, they, and the gap that separates them, will be permanent. If they are separated physically but not ecologically, they will be permanent unless at some future time the two populations are reunited. We do not consider this possibility because species should not be defined by hypothetical future events that may, or may not, happen [19].

It should be noted that although the underlying theory was described by Barraclough *et al*. [12], the authors avoided using the word “species” to describe the long-lasting clusters formed by physical isolation or adaptation. This had the unfortunate effect that some readers failed to recognize that these clusters are fundamentally different from clusters formed by drift alone and are, in fact, species under the evolutionary and general lineage species concept.

To assign individuals to species, one needs an operational species criterion. A species criterion for our model of speciation must solve two problems, both of which are fundamentally statistical in nature:

1. It must identify inclusive populations separated from each other by gaps that are significantly greater than 2*N*_e_ generations deep.
2. This must be done using very small samples of individuals from populations that are often very large.

**Figure 2:**
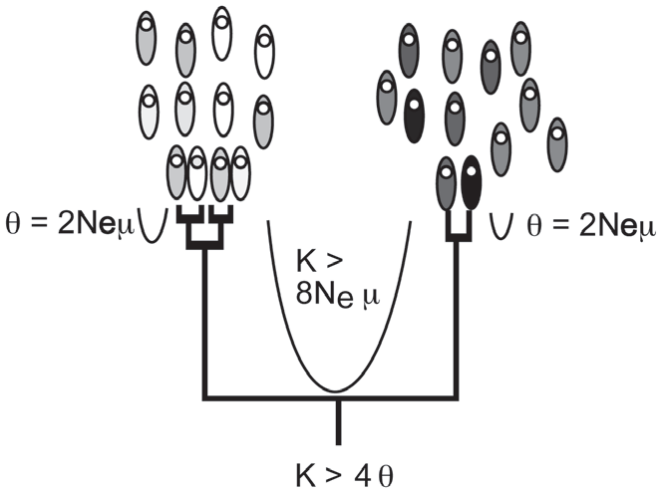
Using phylogenetic analysis and the 4× rule to identify species. If phylogenetic analysis shows that small samples from two populations are reciprocally monophyletic, *and* if the mean sequence difference between them is more than four times θ = 2*N_e_*μ estimated from the within-sample variation, then the samples came from different species.

The theory in our speciation model suggests several possible species criteria, one of which is the 4× (“4 times”) rule that we developed to find species in asexual and clonal organisms using DNA sequences (Fig. 2) [6], [7]. From basic population genetic theory (e.g. [20]) the mean sequence difference between individuals in a clade, called the nucleotide diversity π, is an estimator of θ≈2*N*_e_μ, where *N*_e_ is the effective population size and μ is the mutation rate per base pair per generation. More precisely, θ = π/(1–4π/3) which is very close to π when 2*N*_e_μ is small. Note that this effective population size includes the effects of selection, notably periodic selection and background selection, which are important in reducing the effective size of asexual populations. Moreover, the mean sequence divergence *K* between individuals in two candidate clades is an estimate of 2*t*μ, where *t* is the time to the most recent common ancestor of the clades. Here *K* is the observed sequence difference *D* corrected for multiple hits. We want to identify clusters that are separated by *t≥*4*N*_e_ generations, which is the upper 95% confidence limit of the coalescent time and of the depth of gaps formed by random drift. The gaps between these clusters have a probability of less than 5% of being formed by random genetic drift within the inclusive population that is an evolutionary species. The clusters will appear as clades in a phylogenetic tree. For such clades, the ratio *K*/θ = 2*t*μ/2*N*_e_μ≥8 *N*_e_μ/2 *N*_e_μ = 4. Hence two clades separated by *K*≥4θ represent populations that have diverged by at least 4*N*_e_ generations. When θ is small, the rule is closely approximated by *K*≥4π. Note that *N*_e_ and μ cancel in the derivation, an especially nice feature because these two parameters are almost always unknown and difficult to estimate.

The process of speciation and forming gaps deeper than those produced by stochastic processes also causes lineage sorting leading ultimately to reciprocal monophyly of the independently evolving populations. When *K*≥4θ, the two populations will be reciprocally monophyletic with probability ≥95%. Remarkably, the 4× rule can infer reciprocal monophyly of entire populations from extremely small samples of individuals ([21] Fig. 6ii). If two samples of size ≥2 are reciprocally monophyletic, there is at least a 95% probability that the populations are also reciprocally monophyletic after 4*N*_e_ generations. The surprisingly high statistical power of small samples comes about because the probabilities are conditioned on the samples themselves being reciprocally monophyletic.

Thus it is possible to recognize asexual species by sequencing any suitable gene (“suitable” is discussed below) from a number of individuals, making a phylogenetic tree of the sequences, and testing well-supported terminal clades for adherence to the 4× rule. Clades that (i) are well supported by phylogenetic analysis, and (ii) pass the 4× test *and* are basal or simple, containing no such clades within them, are inferred with high statistical confidence to be samples from independently evolving populations; in other words, evolutionary species. We have used 95% confidence intervals for identifying species within samples of bdelloids because this probability is widely used in biology, but one could choose a more conservative criterion such as 99% confidence that two populations are reciprocally monophyletic. This would require either somewhat larger sample sizes or the use of, e.g. a 4.5× or 5× rule (Figure 6 of [21]. The general procedure of using the K/θ ratio to estimate the probability that two samples are from different evolutionary species can be called the K/θ method. For simplicity, we will continue to refer to the procedure as using the “4× rule” when the 95% confidence limit is used. To emphasize that this criterion is explicitly grounded in population and evolutionary genetic theory, we will refer to the species detected by the 4× rule as evolutionary genetic (EG) species.

Previously the 4× rule was applied to a sample of 110 bdelloid rotifers collected mainly in the U.S., finding 21 species clusters and 19 singlets [6]. This was the first demonstration that asexual organisms undergo speciation to produced evolutionary species corresponding to a rigorous theoretical model. We showed that at least some of these species are adapted to different niches: some species are sympatric; two were shown to have different temperature tolerances; and some of the species belong to different genera that differ consistently in thei manner of feeding and resource utilization. Subsequently, a different method of phylogenetic analysis, the GMYC method, was used to demonstrate independently evolving populations within a bdelloid genus [22] and within a single bdelloid morphological species [23]. The application of the 4× rule in our earlier paper differed slightly from the one used in the present paper in that we used nucleotide diversity directly as an approximate estimate of 2*N*_e_μ. However, as noted above this difference is insignificant when the nucleotide diversity is small.

Here we apply the 4× rule to detect additional species in a much larger and more diverse sample of bdelloid rotifers; show that these species are not artifacts of inadequate sequence length or sampling; and show that although the 4× rule is based on pairwise comparisons of clusters, it reflects an underlying general pattern of diversification. We further demonstrate the generality of our speciation model by successfully applying the 4× rule it to other asexual taxa: oribatid mites; the oligochaete worm *Lumbriculus*; the fungus *Penicillium*; and several groups of protists. Each group is shown to have diversified into a number of EG species, which in some cases were already predicted to exist on the basis of subtle phenotypic differences or biogeographic evidence.

## Results

### Bdelloid rotifers

We applied the 4× rule to the cloned progeny of 226 individual female bdelloids collected from 56 sites representing a variety of habitats from sea level to timberline in 11 states in the continental U.S., Hawaii, and one location each in Australia, Italy, and the Netherlands. DNA was isolated from each isolate and 591 bp (including 196 complete codons) of the mitochondrial *cox1* gene encoding cytochrome *c* subunit 1 were amplified and sequenced [6]. Mitochondrial genes were used because they are effectively haploid [24], which eliminates the paralogy and high sequence divergence between alleles that can make it difficult or impossible to recover the correct phylogenetic tree using nuclear gene sequences in bdelloids [10], [11]. Moreover, one can directly sequence the uncloned amplification products from haploid genomes.

**Figure 3:**
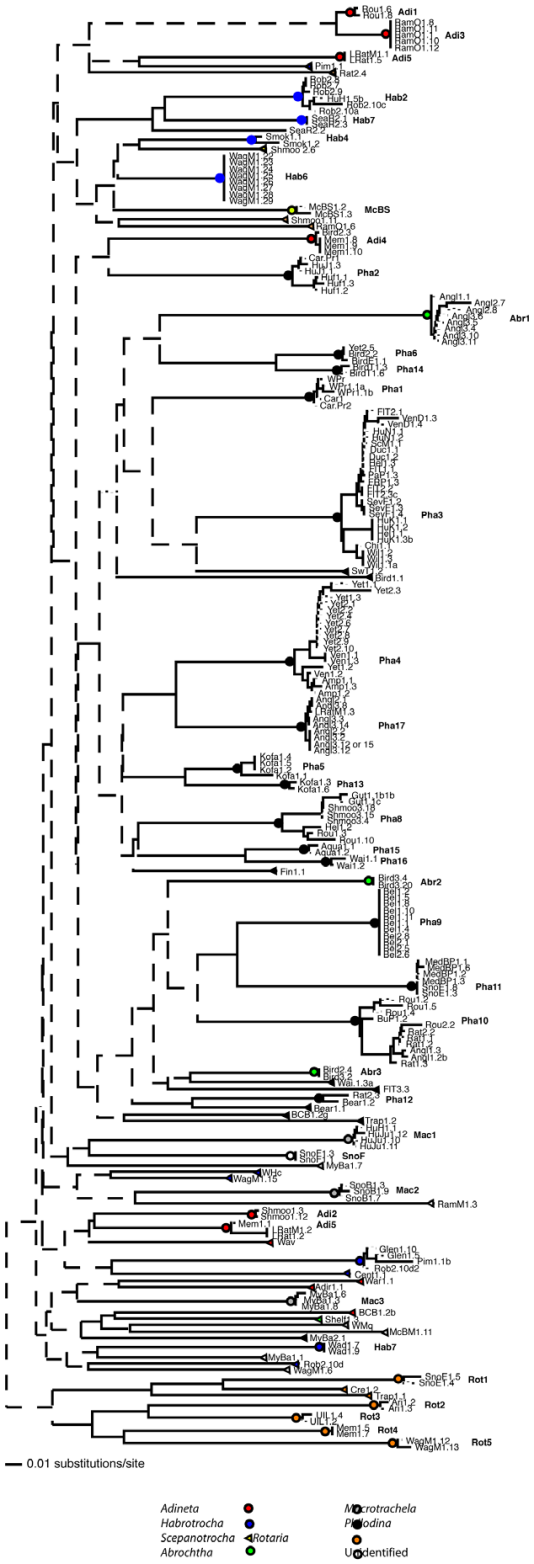
Bdelloid rotifer: Maximum Likelihood tree made with PAUP using the procedure described by Hall [51]. The tree was rooted with clones of *Rotaria*, because several analyses support a basal position for this genus. Pairwise distances were corrected for multiple hits using a general time reversible model with site-specific substitution rates for the first, second, and third codon positions. Colored circles and triangles respectively indicate clades and singlets that were identified as species using the procedure described in the text. Pending formal species descriptions, these species are given temporary names consisting of the abbreviated genus and a number. Adi = *Adineta*; Hab = *Habrotrocha*; Sce = *Scepanotrocha*; Abr = *Abrochtha*; Mac = *Macrotrachela*; Rot = *Rotaria*; Pha = *Philodina*. Species labeled McBS1 and SnoF1 were not identified and are named after their sources (McBeth Spring and Snowy Range site F). Dashed lines were supported by <50% of 1000 neighbor-joining bootstrap replicas, each with 10 random-addition replicates.

Phylogenetic analysis of the aligned *cox1* sequences was done for all clones. Of the 591 sites (base pairs) of this gene, 469 are variable and 378 of these are parsimony-informative. Figures 3 and S1 show a maximum-likelihood tree in which a number of clades are supported by at least 70% of 1000 bootstrap replicas. The same clades were strongly supported by bootstrapped phylogenetic analyses using parsimony, or neighbor-joining with sequence differences corrected for multiple hits with the parameter-rich maximum likelihood models selected by ModelTest [25], or corrected with the one-parameter Jukes-Cantor model (data not shown). These clades were also found when only the third codon position was used to emphasize neutral substitutions, as expected since the great majority of substitutions are synonymous (data not shown). The only exception was the clade consisting of clones Kofa1.1, 2, 4, and 5, which received 59–68% bootstrap support in neighbor-joining trees made with the GTR+I+G model.

Of the clades strongly supported by phylogenetic analyses, 41 satisfy the 4× rule and are therefore samples from different EG species. These evolutionary genetic species are shown in Fig. 3. Most were identified to genus. A few have been shown to belong to already-describe species or new species, but most have only a temporary name (Supporting Information Table S1).

#### Bdelloid Species Are Not Artifacts of Incomplete Sampling

**Figure 4:**
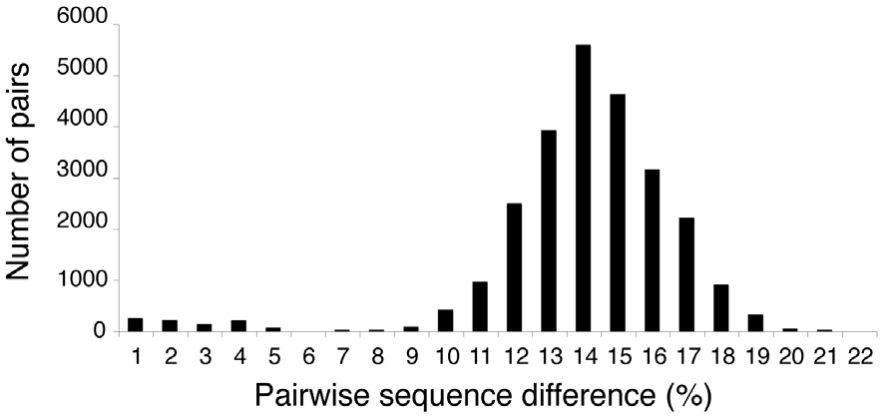
Bdelloid rotifers: frequency distribution of uncorrected pairwise sequence differences, in bins of 1%. The differences within species are not corrected for multiple hits, which is appropriate because all are less than 10%. The differences between species are corrected for multiple hits using the TVM+I+G model selected by ModelTest [25].

Several lines of evidence show that the phylogenetic pattern of shallow clusters of very similar clones separated by deep gaps is not likely to be the consequence of incomplete sampling:

First, an asexual lineage descended from a single individual without divergent selection or geographic isolation is expected to show an exponential distribution of pairwise sequence differences. Incomplete sampling, like stochastic variation in reproduction among lineages, will make the distribution less smooth but will not produce deep gaps [26]. In contrast, the pairwise distribution of sequence differences among our clones is strongly bimodal (Figs. 4 and S2). The first mode represents mainly sequence differences between individuals within species, with an expected mean of 2*N*_e_μ differences per site. The second mode represents differences between species, with an expected mean ≫ 2*N*_e_μ differences per site. The reality of the two modes is supported by the observation that the mean uncorrected sequence difference *D* within species is significantly smaller than *D* between species. The clear distinction between the two nodes may be partly due to strong hitchhiking and background selection, which will tend to decrease the effective population size *N*_e_ for a given census size *N*, thereby hastening the coalescence process and reducing diversity within species. Note that this is a feature of all asexual organisms and should make the application of the 4× rule easier by eliminating some ambiguous cases.

The second line of evidence that the species are not artifacts of incomplete sampling is that as more animals were sampled from more diverse sites, none of the gaps were filled in and no EG species were fused. Of the 41 species, 8 were observed when the sample size reached 27 bdelloids, taken in 14 collections from 13 sites. Two of the collections (War1 and Car1) were obtained from biological supply houses which had no information about the source or history of the collections; Car1 had isolates appeared in two clades and may have included animals from more than one site. Of the remaining 12 collections, 1 came from Columbus, Ohio (Gut1); 5 came from sites close together in Hueco Tanks State Park, Texas (Huf1, HuJ1, HuK1, HuN1); and 6 were from 5 locations in southern Arizona. Subsequently our sample size increased 8-fold and the number of sites increased more than 4-fold, including animals from many more habitats in the continental U.S., Hawaii, Europe, and Australia. Three sites were sampled three times in different years, five sites were sampled two times, and the remainder once each; samples were taken at all seasons. Phylogenetic analyses were done repeatedly during this process and EG species were identified. While the number of species and singlets increased as the number and diversity of collecting sites increased, at no point were two previously identified species joined. Instead, each new isolate joined with a singlet to form a new species, or joined an existing species, or became a new singlet.

These results are strong evidence that few, if any, of the EG species are artifacts of inadequate sampling of individual sites, habitats, or geographic locations. Of course the future addition of new isolates might cause two species to be joined, thereby fusing two evolutionary genetic species into one; this might happen, for example, to Pha5 and Pha13, which are very closely related. It is also possible that a large diverse species such as Pha3 might be split into two species. But lumping and splitting of species are commonplace in systematics and can happen with species defined by any criterion. We suspect that evolutionary genetic species may be more stable than many others because of the rigorous theoretical basis and statistical power of the 4× rule.

#### Most or All of the Clades that Satisfy the 4× Rule are Species, Not Higher Taxa

As we noted above, the simplest explanation of the bimodal distribution of pairwise sequence differences is that the small differences separate individuals within a species, while the large differences are between species. The same phenomenon can be seen when the logarithm of the number of lineages in an ultrametric tree is plotted against time: the number of lineages shows a dramatic increase in the latest time period (data not shown). This indicates a recent switch to a much higher rate of lineage diversification where species lineages are replaced by individual lineages. The rate of lineage branching within a species is on the order of magnitude of generations, while the rate of branching among species is determined by rates of speciation and extinction, which are on the order of thousands of years or more. Highton [27] found a bimodal frequency distribution of pairwise sequence difference, and bi- or multi-modal distributions of Nei's pairwise *D* from allozyme data, in Plethodontid salamanders. He attributed the mode with the smallest distances to variation within species, as we do. Acinas *et al*. [9] reported distinct clusters of bacterial rRNA sequences in a large sample of environmental sequences, and showed a similar increase in branching rate at recent times. They noted that such clusters would be formed by periodic selection for adaptation to new niches as proposed by Cohan [28]. Pons *et al.* [29] and Fontaneto et al. [22] used maximum likelihood analysis to detect increasing branch rates at the species/individual borderline in sexually-reproducing tiger beetles and bdelloid rotifers, respectively.

An alternative explanation would be that most species underwent a drastic increase in net speciation rates at about the same time. This might happen if there was a mass extinction followed by an adaptive radiation in most or all of the surviving lineages. In this case we might expect to find a third peak of pairwise sequence differences representing the individuals within species. In fact, Fig. 4 shows that there is some indication that the within-species range (<4%) is itself bimodal with a gap at about 1.5%. Both of these modes consist almost entirely of distances within species, with one exception: Adi1 and Adi3 are very closely related, differing by 3.6%. They are samples from different evolutionary species by the 4× rule, but they are so similar that additional sampling could easily combine them. 26 species fall only in the lower mode; 11 fall in both modes; and 3 fall only in the higher mode. Of the within-species differences in the higher peak, more than half are due to the species Pha3 and Pha4, with additional significant contributions from Pha10, Pha8, and Pha2 (in order of decreasing importance).

While it is possible that the higher of the two within-species peaks represents higher taxa, presumably genera, it seems unlikely that all of the eight clades that contributed to the second peak underwent nearly simultaneous speciation. A second possible explanation is that Pha3 and 4, or some other clades, were misidentified as species. The 4× rule identifies species using the 95% confidence interval, which means we could err as much as 5% of the time when the ratio K/θ is equal to, or slightly larger than 4. However, the clades with the larger θ values all had K/θ ratios substantially greater than 4 and/or large samples, making this kind of error unlikely. A third possibility is that these clades with large nucleotide diversities are samples from large metapopulations with strongly differentiated local populations. The second peak would represent differences between subpopulations that began evolving independently too recently to satisfy the 4× rule. We suggest that if this is verified by independent evidence, these might be called subspecies.

A possible source of error in species delimitation is that the 591-bp *cox1* gene segment has insufficient information to resolve closely-related species. To test this, we amplified and sequenced a 353-bp region of the mitochondrial *cob* gene encoding the apoprotein of cytochrome *b* from 97 specimens, including ≥2 members of 15 different species. The concatenated sequences found the same species as did *cox1* by itself, indicating that it has sufficient information to resolve species.

Finally, we note that the nucleotide diversities of our proposed evolutionary species, i.e. the mean pairwise sequence differences within the species, range from 0 to 2.4% and average 0.6%. This is within the range of nucleotide diversity seen in other invertebrates as summarized in [30], [31].

#### Most or All of the Singlets Represent Additional Species

Besides the 41 clades that we identify as evolutionary genetic species, the tree of bdelloid *cox1* sequences also contains 30 singlets. We cannot rule out the possibility that future additions to the tree will combine some of these into a clade that obeys the 4× rule. However, all of the singlets are separated from each other and from SG species by pairwise differences that fall within the larger mode of the bimodal distribution of pairwise sequence differences. Also Rosenberg [21] showed that if a sample of one individual from one population and two or more from another meet the 4× test, the probability that they represent reciprocally monophyletic populations is ≥94%, only slight lower than for samples of two from each population. Thus the singlets are probably sampled from additional species. This raises the number of bdelloid species detected in our sample to 71.

#### Comparison of Evolutionary Genetic Species to Described Morphological Taxa

The bdelloid rotifers have been divided into 4 families and 20 genera on the basis of discrete character differences (e.g. presence or absence of stomach lumen; presence or absence of ciliated trochal lobes; 0, 2, 3, or 4 toes; number of major teeth on the trophi) [32], [33]. If the species identified by sequence analysis were the same as those identified by morphology, we would expect that none of the evolutionary genetic species would contain individuals that could be assigned to two or more genera on the basis of morphology. 218 of the clones were identified to genus (Supporting Information Table S1); no evolutionary genetic species includes members of more than one genus. Note, however, that the *cox1* sequence alone is not sufficient to determine whether the genera are monophyletic.

In addition to the 20 genera, bdelloid rotifers have been divided into 382 species on the basis of phenotype [33], [34], [35], [36]. Many of the characters used to differentiate between species are qualitative and subjective. We attempted to identify the members of two genera in our sample to species, using [32] as reference. The three species and one singlet of *Abrochtha* are markedly different in morphology from both of the previously described species. Three of these new species are quite similar in external morphology, but can be distinguished by the shape or size of their trophi (Diego Fontaneto, Giulio Melone, and Claudia Ricci, personal communication). All five of the species of *Rotaria* that are represented by two individuals each also differ in morphology from all of the 24 described species of this genus (the two singlets were lost after before they could be identified to species). Formal descriptions of these hitherto-undescribed species have been submitted for publication.

#### Other Bdelloid Data

Fontaneto et al. [22] used the general mixed Yule coalescent (GMYC) model of Pons et al. [29] to find 13 “independently evolving entities” in a collection of 76 specimens of the bdelloid genus *Rotaria*. We used Neighbor-joining in PAUP to make a vphylogenetic tree of 73 of their *cox1* sequences (GenBank accession numbers DQ656809-DQ656882); we corrected for multiple hits with the best-fit evolutionary model selected by Model Test [26] (the general time-reversible model plus invariant sites and a gamma distribution of substitution rates). The tree is shown in Fig. S3. We applied the 4× rule to the corrected and uncorrected pairwise sequence differences and found that all 13 of the “entities” are evolutionary genetic species. 28 singlets in their collection are probably represent another 28 EG species. The frequency distribution of pairwise sequence differences is bimodal, as it is in our data set that includes 10 *Rotaria* plus 210 individuals from six additional genera. Fontaneto et al. assigned all of their *Rotaria* specimens to previously described morphological species, but only *R. socialis* and *R. citrina* were EG species; each of the other morphospecies included two or more EG species.

### Oribatid Mites

Orabatid mitres (Acari, Oribatida) include a number of ancient parthenogenetic groups including the Camisiidae which are represented in Cretaceous amber from 85Mya. Heethoff et al. [37] sequenced a segment of the *cox1* gene from 65 specimens of *Platynothrus peltifer*and 4 specimens of *Nothrus silvestris*. Their phylogenetic analysis revealed a pattern of shallow clades separated by long branches, similar to that of bdelloid rotifers. They found a single clade of *N. silvestris* but *P. peltifer* fell into seven distinct clusters with nucleotide diversities <2%. The seven clusters were separated by deep branches and also by geography; the authors suggested that the clusters might be different cryptic species separated by continental drift and mountain uplift. Molecular clock estimates put the separation of *P. peltifer* and *N. silvestris*, and thus the origin of the parthenogenetic Camisiidae, at 110 Mya and the initial diversification of the *P. peltifer* species at 15–58 Mya.

We applied the 4× rule to the *Oribatid* mite sequences of Heethoff et al. [37] using a sequence file kindly provided by Michael Heethoff. The GenBank accession numbers for these sequences are DQ381157–DQ381226. We made a frequency distribution of the pairwise sequence differences between the oribatid specimens as described in Materials and Methods, and found a bimodal distribution of pairwise sequence differences (data not shown). Application of the 4× rule to the pairwise differences verified that *N. silvestris* and the seven clades of *P. pelitifer* are all EG species. We used uncorrected differences only; because the within-species differences were too low to be significantly affected by multiple hits and the between-species differences were so much larger that there was no ambiguity about the species boundaries.

### Oligochaete Worms

Asexual reproduction is common in oligochaete worms. We used the 4× rule to verify that *Lumbriculus variegatus*, a widespread Holarctic freshwater oligochaete, consists of two evolutionary species. Sexual reproduction has been reported in *L. variegatus* but appears to be rare; usually the organisms reproduce by fragmentation and regeneration. Gustafsson et al. [38] used a Bayesian analysis to show that *cox1* divided this species into two well-supported clades, I and II. These clades were also supported by mitochondrial 16S and nuclear ITS sequences. Clades I and II further differed in ploidy, and were sympatric in the samples from four widely separated bodies of water in Sweden. The authors concluded that these are “separately evolving metapopulation lineages”, i.e. evolutionary species. We verified this by applying the 4× rule to the *cox1* data, using the mean pairwise sequence differences within and between the two clades reported in [38]; the uncorrected mean sequence difference between the clades was 17.7%, while the mean differences within the clades were 0.6% for clade I and 1.3% for clade II. Using the probability tables provided by Noah Rosenberg as described above for bdelloids, we found that the probability that clades I and II are samples from a single population, i.e. that they belong to the same EG species, is much less than 0.005, given the sample sizes of 23 and 7 for clades I and II respectively. Note that if the differences between the clades had been corrected for multiple hits, the between-clade difference would have been even larger and the evidence for independent evolution of the clades stronger.

### Fungus *Penicillium*

Members of the fungus genus *Penicillium* have no known sexual stage, but are polyphyletic with the genus *Eupenicillium* for which sexual stages are known. Seifert et al. [39] used a very large dataset of *cox1* sequences from isolates of *Penicillium* plus outgroups (GenBank accession numbers EF180096 through EF180449). The *cox1* gene is less diverse in these fungi, leaving many taxonomic relationships unresolved; consequently we limited our analysis to the data that are included in the bottom half of the tree in Fig. 2 of Seifert et al. [39] (the top half contained many more poorly-supported clades). The reduced data consist of *cox1* sequences from 188 specimens assigned to 35 named species of *Penicillium*; the closely related sexual species *Eupenicillium osmophilum*; and the outgroups *Aspergillus niger*, *Talaromyces flavus*, and *Talaromyces*. *trachyspermus*.

We tested this data set for evolutionary genetic species, using aligned sequences provided by Keith Seifert. We trimmed the sequences to the 545-bp coding region common to all specimens and made a bootstrapped phylogenetic tree using neighbor-joining without correcting for multiple hits; Fig. S4 shows the tree with evolutionary species indicated. We limited our analysis to clades that are reciprocally monophyletic with ≥70% bootstrap support and have at least 3 members. Application of the 4× rule to these clades showed that 23 of the *Penicillium* clades identified as different species by systematists using mainly phenotypic data qualify as evolutionary genetic species. Four other evolutionary genetic species are clades, each of which consists of two or three phenotypic species that cannot be distinguished by the 4× rule. The phenotypic species within an evolutionary genetic species could be subspecies that have begun but not completed the process of speciation; alternatively they could be evolutionary genetic species that cannot be distinguished because *cox1* is insufficiently variable. Two of the described species each form a single robust clade but are not evolutionary species. A sample of the results is shown in Table S2; the complete analysis is available from the senior author on request. Although further analyses are clearly needed, these results suggest that *Penicillium* has undergone extensive divergence to produce independently evolving species.

### Heterotrophic Marine Flagellates

Scheckenbach et al. [40] did a phylogenetic analysis of SSU rDNA sequences of 31 individual heterotrophic marine flagellates belonging to five morphospecies with cosmopolitan distribution. These microbial eukaryotes belong to the putatively asexual groups Apusozoa, Bicosoecidae, and Bodonidae. The authors concluded that two or three of the five species studied (*Ancyromonas sigmoides*, *Caecitellus parvulus*, and possibly *Rhynchomonas nasuta*) could be divided into more than one cryptic species, seen as clades of individuals with very similar sequences, separated from each other by sequence differences that appeared too great for intraspecific variation. However, no specific species concept or criterion was used. Tables 3 through 7 of Scheckenbach et al. [40] give the uncorrected pairwise sequence differences between the SSU rDNAs of specimens of *Rhynchomonas nasuta*, *Amastigomonas debruynei*, *Ancromonas sigmoides*, and *Cafeteria* species, respectively. We used these uncorrected differences to calculate the nucleotide diversity π and θ = 2*N*_e_μ; with this information and the sample sizes for each species we found the probabilities that they were samples from independently evolving populations in Rosenbereg's table. The results (Table S3) showed that the cryptic species in *A. sigmoides*, *Cafeteria* spp., and *C. parvulus* are evolutionary species with probabilities >0.99. The probability that the two clades of *R. nasuta* samples from different species lies between 0.94 and 0.95, almost but not quite statistically significant. It is likely that additional sequence data, more specimens, or the use of a more variable locus would clearly separate *R. nasuta* into two cryptic evolutionary species.

We agree with the authors that the three isolates of the sixth morphospecies, *Amastigomonas debruynei*, cannot be separated into species. However, this study used SSUrDNA which is generally believed to be less variable than *cox1* and other sequences commonly used to find species. Possibly a more variable molecule or a longer sequence would find species within *A. debruynei* and possibly subdivide the other morphospecies further. Nevertheless, it is important to note that even if the independently evolving populations detected with the 4× rule are not species but clades of closely related species, their detection demonstrates past speciation events in putatively asexual protists.

### Chlorophyte Alga *Ostreococcus*

The smallest eukaryotic cell is an apparently asexual photosynthetic marine chlorophyte alga described as a single species, *Ostreococcus tauri*. The small size and simple structure of this organism has prevented any morphological discrimination among isolates, but SSU rDNA sequences fall into four different clades [41]. Rodriguiz et al.[42] sequenced the ITS1, 5.8S, and ITS2 regions of 23 individuals isolated from different marine environments. Grimsley *et al.* [43] showed that *Ostreococcus* are haploid in culture and probably in nature, and although they have some form of outcrossing and recombination the frequency is low; consequently θ≈2*N*_e_μ and the theory given above for organelle genes in asexual organisms should apply to these data.

A maximum likelihood phylogeny showed that the ITS sequences of fell into three clades (A, B, and D) and a singlet (C).We repeated the phylogenetic analysis, using neighbor joining and the same evolutionary model and parameters used by Rodriguiz et al. and the aligned sequences kindly provided by Francisco Rodríguez, and found the same tree structure shown in their Figure 1. We used the pairwise sequence differences to estimate the nucleotide diversity π and θ for each clade and the mean pairwise differences K among the clades and the singlet. From these data we found the probability P that the clades are evolving independently of each other, using the probability table provided by Noah Rosenberg. The results, summarized in Table S4, allow us to infer with probability greater than 0.99 that the clades A, B, and D and the singlet C are samples from four different evolutionary genetic species. As with the heterotrophic algae, there is no way of knowing if additional data or more variable regions would detect species within these four independently evolving groups, but the great difference between the diversity within and between the clades suggests that this is unlikely.

## Discussion

Our results show that a simple species criterion with a rigorous theoretical basis in population genetics and statistics, the 4× rule, can be used to detect species in even small samples of individuals from a variety of asexual organisms. We confirm the results of Birky et al. [6], who first showed that an asexual lineage, the bdelloid rotifers, have undergone extensive speciation without sex. We also show that speciation is a general phenomenon in asexual organisms by applying the 4× rule to a very diverse collection of organisms: another group of invertebrates (oribatid mites) fungi, green algae, and heterotrophic flagellates. In the bdelloids, there is strong evidence that the species are not artifacts of inadequate sampling or sequence length.

The bdelloid and oribatid studies use the same *cox1* sequence as the Barcode of Life project. However our approach is very different from most barcoding, which attempts to use DNA sequences to identify species already defined by traditional systematics. Barcode identification of a species is based on empirically-determined limits of sequence differences, and is usually not justified by any theory. Moreover, organized barcoding efforts have focused on sexual organisms in which the gene used to assign individuals to a species may behave differently from the majority of genes, including those involved in speciation, and can fail to make a proper assignment in many cases [44]. In contrast we use a theory-based species concept and criterion to define species as well as to assign individuals to species, in asexual organisms where the gene sequence tracks the genealogy of the whole organism and its genome.

Our 4× rule is not a fixed threshold; it is the ratio K/θ of measurable parameters which can be greater or less than 4, depending on the observed values of sequence diversity and on the sample size. However, it is a conservative way of delimiting species with at least 95% statistical confidence for all but the smallest samples. In particular, the 4× rule is not to be confused with the “10× rule” proposed by Hebert et al. [45] for use with DNA barcoding. That rule says that the sequence difference between two species should be at least 10× the sequence difference within either of those species. This is an empirical rule based on barcoding data from sexual species defined by traditional systematic methods. The 4× rule is also conceptually distinct from the 10% sequence divergence rule sometimes used to delimit bacterial species.

Our approach uses standard statistical methods to account for finite sample sizes of individuals in the identification of sister groups, the calculation of θ, and in inferring θ/K (Methods). It does not account for the finite sequence length used to estimate π and hence θ. That the addition of *cob* sequences did not change species assignments in the bdelloid data argues that *cox1* by itself has sufficient information, at least for this group. A notable and attractive feature of the 4× rule is that, although the derivation involves two parameters, *N*_e_ and μ, that are difficult to measure, they cancel and the application of the rule depends only a ratio.

We cannot rule out the possibility that additional samples of organisms or the addition of more sequence data will show that some of the species identified by the 4× rule are actually higher taxa, but the addition of new data can force systematic revision of species identified by any method. We predict that although some splitting of evolutionary genetic species may occur in the future, lumping of these species is extremely unlikely in the bdelloids, the oribatid *Platynothrus*, the green alga *Ostreococcus*, and the heterotrophic algae *Cafeteria* and *Caecitellus* because of the long branches that separate the species. Of course if speciation events occur too rapidly (on the order of every 4*N*_e_ generations or less), the 4× rule cannot distinguish species. But all species criteria are unreliable or fail outright in this case. Also the sequences used to distinguish species must have sufficient variation; the *cox1* sequence is probably not sufficiently variable in to distinguish many species in *Penicillium*. An alternative explanation is that some lineages of this fungus may be undergoing very rapid speciation (an adaptive radiation or a period of rapid dispersal followed by geographic isolation).

Speciation is often a gradual process [14], [30], [46], beginning with whatever event initiates it and culminating in reciprocal monophyly. Clearly it is possible to apply the ratio K/θ and the statistics of Rosenberg [21] to any two sister clades to obtain a quantitative measure of the probability that they are samples from distinct species. Likewise the procedure could be used as a quantitative measure of the progress of two populations along the pathway from varieties to species, bearing in mind of course that speciation may never be achieved.

We propose to limit the term “species” to inclusive populations that can be shown with at least 95% confidence to be separated by gaps deeper than 4*N*_e_ generations. Species that do not meet this criterion but are in the process of speciation as shown by adaptation to different niches or allopatry (preferably long-term) should be called subspecies. Our approach differs from much of traditional taxonomy in that we do not believe that populations must have distinguishable phenotypes before they can be assigned to different species. There is no reason why the definition of species should depend on our ability to detect the sometimes subtle or unexpected differences that cause organisms to evolve independently, and the differences that we can detect may have nothing to do with evolutionarily independence. The limited ability of human beings to find adaptive phenotypic differences also leads us to believe that two populations evolving independently because they have been physically isolated more than 4*N*_e_ generations should be considered different species even if there is no evidence, morphological or otherwise, of their adaptation to different niches. Although species could, in theory, become sympatric and fuse at some time in the future, it is also possible that the isolation may persist indefinitely and in fact will lead to ecological differentiation in the future. It is also possible that one or both species will become extinct without ever fusing. Species delimitation should rest on observations, not on hypothetical future changes in the ranges of populations.

It is worth noting that the data of Fontaneto et al. [22], [23] show that the traditional morphological species of bdelloids do not correspond well to evolutionary species as detected either by our 4× rule or by their branch rate method. Sequence analysis divided most traditional species into two or more evolutionary genetic species. Although morphometric analysis of the trophi confirmed the phenotypic distinctiveness of the traditional species, even this objective method of classification by phenotype failed to detect the cryptic evolutionary genetic species. We conclude that the most rigorous approach to the systematics of bdelloids and other asexual organisms is to identify evolutionary genetic species by sequence analysis and then search for discrete morphological differences that could be used for routine classification.

### The Evolutionary Genetic Species are Not Members of One or a Few Metapopulations

Two reviewers of earlier versions of this paper argued that the clusters we call species could be local populations or demes within one or a few species. Each such species would be a metapopulation consisting of two or more local populations connected by migration or by periodic extinction and recolonization. This interpretation is clearly ruled out for the bdelloid rotifers by the depth of the branches separating the species, which has two consequences. First, the effective population size of a single species that includes all or many of the clusters we call species would be on the order of 0.1 to 0.2 as can be seen in Fig. 4, an order of magnitude higher than the norm for invertebrates. Second, the gaps between the clusters have lasted on the order of 4.5 to 9 million years, estimated using a *cox1* molecular clock rate of 2.2% per My [47]. This is longer than the life span of most bodies of water in which bdelloids live, and is a period in which the world in general and North America in particular has undergone major climatic and geological changes which would have erased the barriers between at least some of the hypothetical local populations. Moreover, bdelloids in the anhydrobiotic state are easily dispersed by wind and animals; we previously estimated a dispersal rate of 631 km per one % sequence difference for members of one species [6]. Finally, some bdelloid species are sympatric. The two *Lumriculus* clades are also sympatric and are separated by a similar amount of sequence divergence, presumably also spanning a time when local populations would be repeatedly rearranged, while the cryptic species within the oribatid *P. peltifer* are probably even older. The *Ostreococcus* clades show some differences in habitat (depth), growth rates at different light intensities, and chlorophyll *a/b* ratios, suggesting that they are adapted to different ecological niches. But even without this direct evidence for ecological differentiation, theory shows that while population subdivision may produce transient clusters, it cannot explain the distinct clusters detected by the 4× rule.

### The Evolutionary Genetic Species Criterion and Sexual Organisms

Although we have applied the 4× rule to organisms in which no sexual reproduction has been detected, they should also be applicable with little or no modification to organisms that are sexual but strongly inbred, or predominantly asexual but reproduce sexually infrequently. The theory behind the evolutionary genetic model of speciation is applicable to sexual as well as asexual organisms (Barraclough et al. 2003), and the 4× rule can, in principle, be applied to mitochondrial genes in sexual organisms. In fact, while this manusicript was in review, Marrone et al. [48] used the 4× rule to detect cryptic species within the copepod morphospecies *Hemidiaptomous ingens*. Because the effective population size and the coalescent time is shorter for mitochondrial genes than for nuclear genes, applying the 4× rule to a sexual organism should detect early stages of speciation when the process is not statistically complete for the nuclear genome. The variation in coalescent histories of different genes in sexual species poses special problems that will be addressed in future papers.

### Barcoding Experimental Organisms

Gustafsson et al. [38] point out that *Lmbriculus variegates* is a widely used laboratory organism and the presence of cryptic species raises the possibility that experimental studies in different laboratories may actually have used different species. This is clearly a matter of general concern, since species defined by their phenotype can contain cryptic species differing by thousands or millions of years of evolution. We suggest that barcode sequence(s) of experimental organisms collected in nature should be obtained and archived whenever possible, along with data on the collection site that would enable others to find and use the same species for similar studies. Experimental organisms are often obtained from stock centers or individual collections. However, these stocks can be lost by accident, loss of funding, or loss of individuals with the skill and will to maintain them. Political circumstances can make them unavailable to scientists in other countries. Barcodes and their assignment to evolutionary species would make it possible to recover the same species in the future, as well as determining that stocks in different centers do, or do not, belong to the same species.

## Materials and Methods

Bdelloids specimen sources are shown in Supporting Information, Table S1. Most sampling was opportunistic, but some sites were deliberately sampled more than once and some locations were chosen to represent diverse geography, life zones, and habitats. Specimens were isolated from each collection that contained bdelloids, and each specimen was reared in the laboratory to produce a clone used for identification and DNA isolation. Specimens and their clones were named as described in Supporting Information Table S1. Most clones were identified to genus, based on well-established qualitative morphological and behavioral traits [32], [49]. A few have been identified to species using Donner's key [32]; the published species descriptions are based almost entirely on morphology. Six clones (McBS1.2 and 3; McBM1.11, WagM1.3 and 6, and MyBa2.1) were lost after sequences were obtained but before they could be identified to genus. Generic names, and species names where they are known, are given in Supporting Information Table S1.

DNA isolation used spin columns as described [6], except that Chelex was used in a few cases. Segments of the *cox1* and *cob* genes were amplified and sequenced on both strands as described [6]. All sequences were trimmed to the length of the shortest good sequence (591 bp for *cox1*, 353 bp for *cob*) to ensure that all species were identified using comparable sequences. GenBank accession numbers are DQ078512-DQ078621 for specimens analyzed previously [6]; HMO32919-HMO33023 for new specimens collected by us; and DQ656756:DQ656882 for the *Rotaria* specimens of Fontaneto et al. [22]. Neighbor-joining phylogenetic analyses were performed using PAUP [50] using default settings. Bootstrap support for clades was based on 1000 bootstrap replicas using uncorrected distances as well as distances corrected with the one-parameter Jukes-Cantor model and the parameter-rich general time reversible model with invariant sites and gamma-distributed substitution rates (GTR+I+G) chosen by ModelTest [25]. PAUP was also used in a small parsimony run to get the 50% bootstrap consensus of 236 shortest trees from 7 bootstrap replicas, each with 10 random addition replicas. The maximum likelihood tree was made in PAUP as described in [51].

Application of the 4× rule requires three steps: (i) find statistically well-supported clades using standard phylogenetic distance methods; (ii) for each such clade, estimate nucleotide diversity π by the mean pairwise difference *d* between sequences multiplied by the sample size correction n/(n-1) where n is the number of sequences in the clade; (iii) estimate θ≈2*N*_e_μ by π/(1–4π/3) [52]. When *d* = 0, we used a non-zero estimate of π by assuming that one pairwise difference is 1/*L* where *L* is the sequence length; then π = 2/*Ln*(*n*-1). Finally in step (iv), starting with next-to-terminal clades, test each pair of sister clades with sample sizes *n*_1_≥3 and *n*_2_≥2, or *n*_1_≥5 and *n*_2_ = 1, to see if *D*>4θ; when the sister clades have different values of θ, use the larger. When *n*_1_ = *n*_2_ = 2, we use *D*>4.1θ; and when *n*_1_ = 2–4 and *n*_2_ = 1, we use *D*>4.3θ [21]. When pairwise distances between potential species were close to 4θ, they were corrected for multiple hits to obtain *K* using the GTR+I+G model chosen by ModelTest [25]. For clades that have no well-supported sister clades, we used the smallest *K* to another clade in the polytomy.

## Supporting Information

Figure S1 Full-size Maximum Likelihood tree made with PAUP using default settings.
(2.09 MB TIF)

Figure S2 Frequency distribution of pairwise sequence differences among bdelloids.
(0.03 MB TIF)

Figure S3 Neighbor-joining tree of Rotaria sequences from Fontaneto et al. [22] Tree was made using the GTR+I+G model as described in the text. Colored circles indicate clades identified as evolutionary genetic species using the procedure described in the text.
(0.24 MB PDF)

Figure S4 Phylogenetic tree of *Penicillium*. Neighbor-joining tree of uncorrected *cox1* sequences. Closed circles indicate clades that are evolutionary genetic species as described in the text.
(0.33 MB PDF)

Table S1 List of specimens with phenotypic names, evolutionary species tentative names, collection sites, and GenBank accession numbers.
(0.15 MB DOC)

Table S2 Fungus *Penicillium*. Some examples of evolutionary species identified as described in the text.
(0.03 MB DOC)

Table S3 Heterotrophic flagellates. Four named species each consist of two clades that can be assigned to different evolutionary species with high probability.
(0.02 MB DOC)

Table S4 *Ostreococcus tauri*. Clades A and D, and clades B and C, are pairs of sister clades that can be assigned to different evolutionary species with high probability.
(0.02 MB DOC)
