# Supplementary figures and images for: Using Population Genetic Theory and DNA Sequences for Species Detection and Identification in Asexual Organisms
Source: PLoS One. 2010 May 13;5(5):e10609. doi: 10.1371/journal.pone.0010609 (PMC2869354; doi:10.1371/journal.pone.0010609)

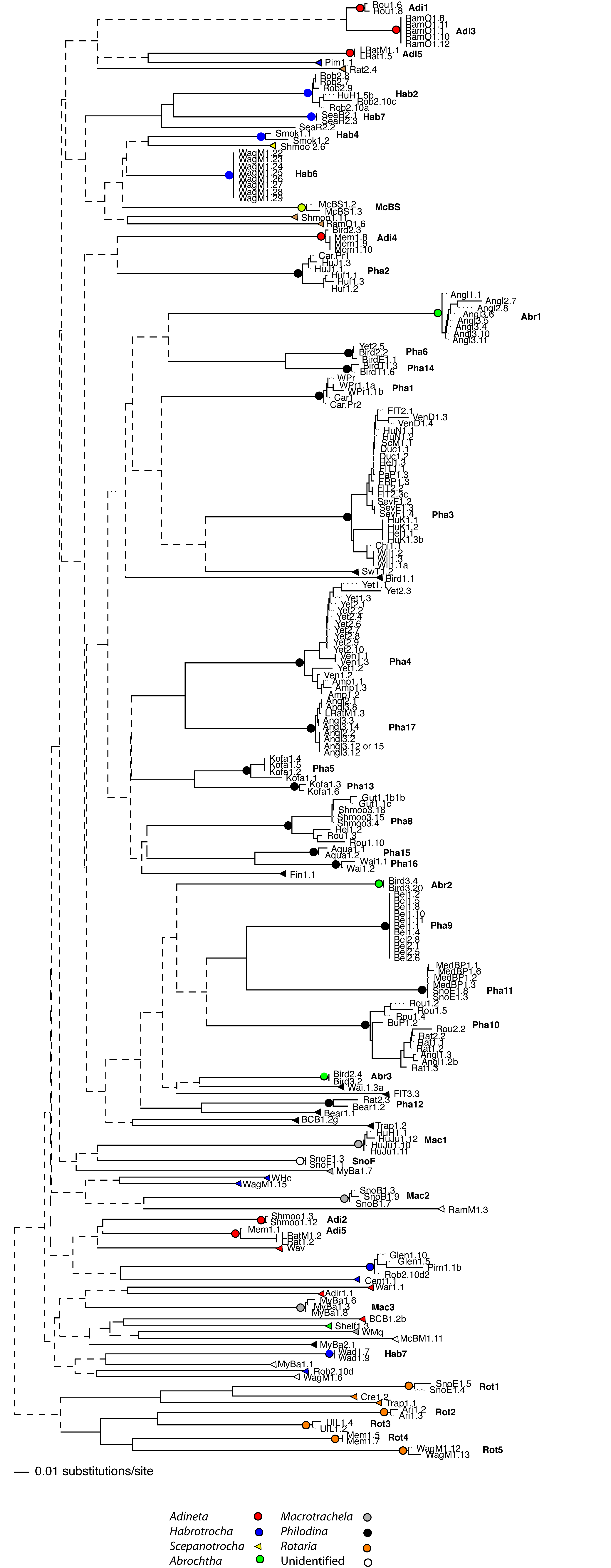

Supplement: Figure S1 — Full-size Maximum Likelihood tree made with PAUP using default settings. (2.09 MB TIF) [file pone.0010609.s001.tif]

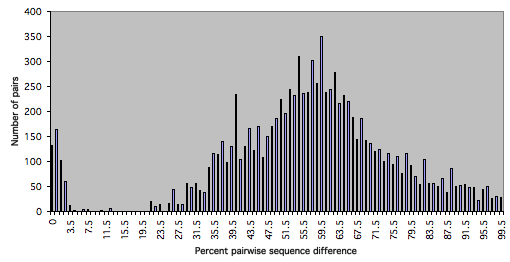

Supplement: Figure S2 — Frequency distribution of pairwise sequence differences among bdelloids. (0.03 MB TIF) [file pone.0010609.s002.tif]

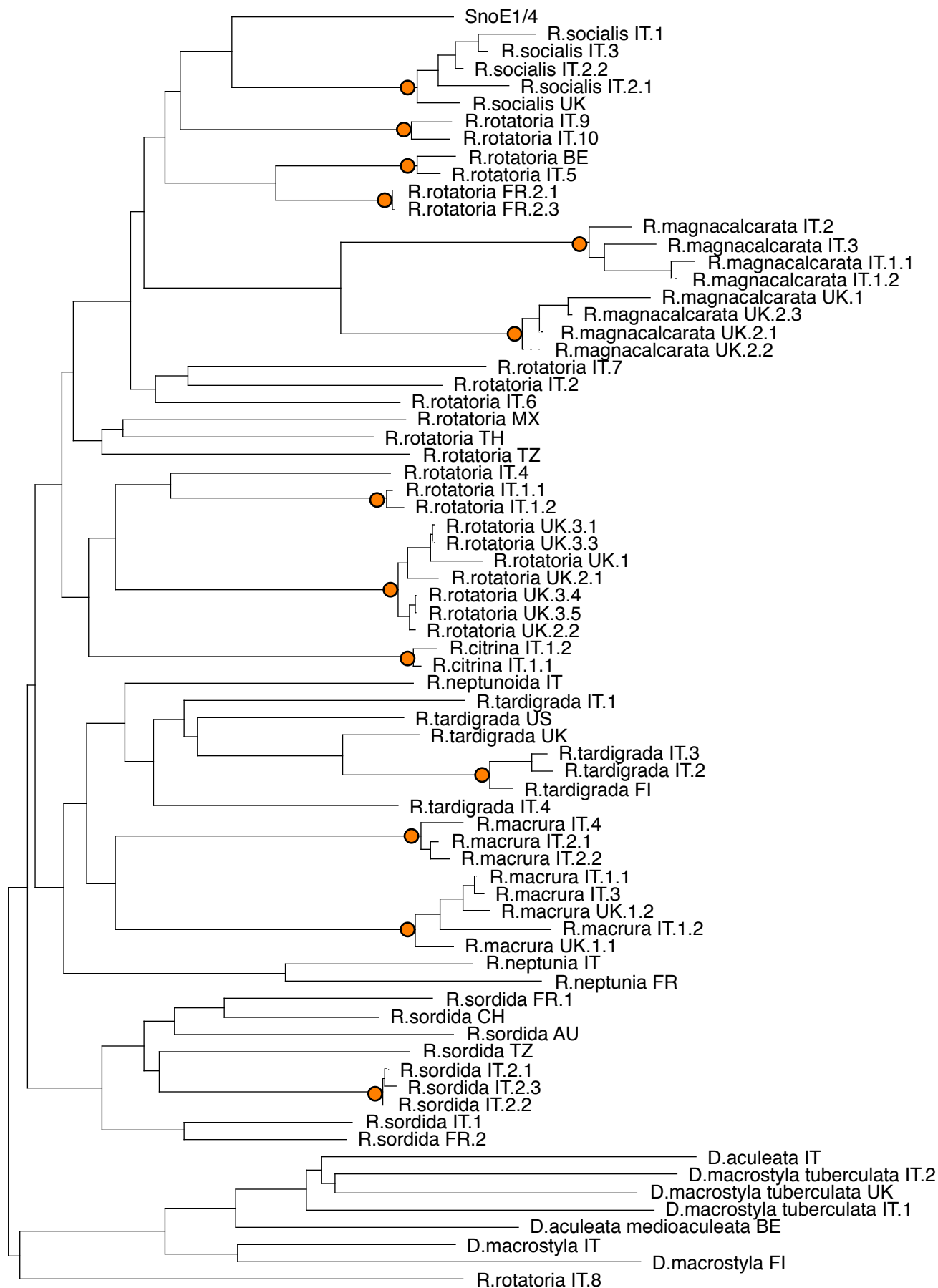

— 0.01 substitutions/site

Supplement: Figure S3 — Neighbor-joining tree of Rotaria sequences from Fontaneto et al. [22] Tree was made using the GTR+I+G model as described in the text. Colored circles indicate clades identified as evolutionary genetic species using the procedure described in the text. (0.24 MB PDF) [file pone.0010609.s003.pdf]

>PSP337 05 101698 Aspergillus niger  
>PSP335 05 55465 Aspergillus niger  
>PSP336 05 12049 Aspergillus niger

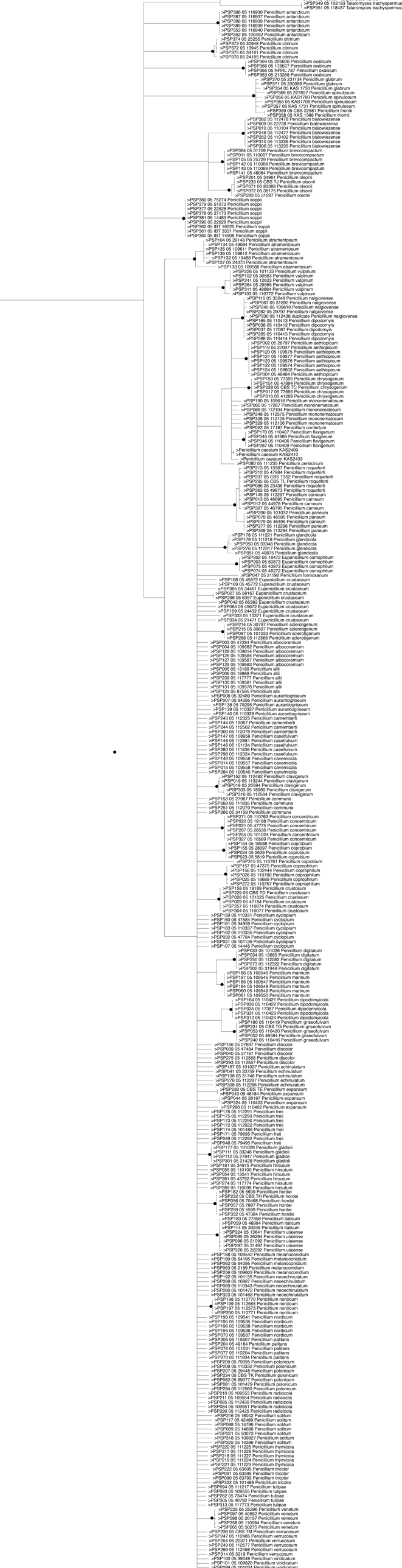

Supplement: Figure S4 — Phylogenetic tree of Penicillium. Neighbor-joining tree of uncorrected cox1 sequences. Closed circles indicate clades that are evolutionary genetic species as described in the text. (0.33 MB PDF) [file pone.0010609.s004.pdf]
