# Supplementary material for: Using Population Genetic Theory and DNA Sequences for Species Detection and Identification in Asexual Organisms
Source: PLoS One. 2010 May 13;5(5):e10609. doi: 10.1371/journal.pone.0010609 (PMC2869354; doi:10.1371/journal.pone.0010609)
Supplement: Table S1 — List of specimens with phenotypic names, evolutionary species tentative names, collection sites, and GenBank accession numbers. (0.15 MB DOC) [file pone.0010609.s005.doc]

# Table S1. List of specimens with phenotypic names, evolutionary species tentative names, collection sites, and GenBank accession numbers.

| Species |  | Clone | Origin | GenBank |
| --- | --- | --- | --- | --- |
| *Abrochtha* sp. nov. | Abr1 | Angl1.1 | Angelfish Pool, Virginia Dale, CO |  |
| *Abrochtha* sp. nov. | Abr1 | Angl2.7 | Angelfish Pool, Virginia Dale, CO |  |
| *Abrochtha* sp. nov. | Abr1 | Angl2.8 | Angelfish Pool, Virginia Dale, CO |  |
| *Abrochtha* sp. nov. | Abr1 | Angl3.6 | Angelfish Pool, Virginia Dale, CO |  |
| *Abrochtha* sp. nov. | Abr1 | Angl3.5 | Angelfish Pool, Virginia Dale, CO |  |
| *Abrochtha* sp. nov. | Abr1 | Angl3.4 | Angelfish Pool, Virginia Dale, CO |  |
| *Abrochtha* sp. nov. | Abr1 | Angl3.10 | Angelfish Pool, Virginia Dale, CO |  |
| *Abrochtha* sp. nov. | Abr1 | Angl3.11 | Angelfish Pool, Virginia Dale, CO |  |
| *Abrochtha* sp. nov. | Abr2 | Bird3.4 | Bird bath, Tucson, AZ |  |
| *Abrochtha* sp. nov. | Abr2 | Bird3.20 | Bird bath, Tucson, AZ |  |
| *Abrochtha* sp. nov. | Abr3 | Bird2.4 | Bird bath, Tucson, AZ |  |
| *Abrochtha* sp. nov. | Abr3 | Bird3.2 | Bird bath, Tucson, AZ |  |
| *Abrochtha* sp. nov. | Abr singlet | Shelf1.3 | Shelf Lake, Snowy Range, WY |  |
| *Adineta* sp. | Adi1 | Rou1.6 | Round Valley, Chniricahua Mts, AZ | DQ078513 |
| *Adineta* sp. | Adi1 | Rou1.8 | Round Valley, Chniricahua Mts, AZ | DQ078514 |
| *Adineta* sp. | Adi2 | Shmoo1.3 | Shmoo Pool, Virginia Dale, CO |  |
| *Adineta* sp. | Adi2 | Shmoo1.12 | Shmoo Pool, Virginia Dale, CO |  |
| *Adineta*sp. | Adi3 | RamO1.1(a) | Ramsey Canyon, Huachuca Mts, AZ |  |
| *Adineta*sp. | Adi3 | RamO1.8 | Ramsey Canyon, Huachuca Mts, AZ |  |
| *Adineta*sp. | Adi3 | RamO1.10 | Ramsey Canyon, Huachuca Mts, AZ |  |
| *Adineta*sp. | Adi3 | RamO1.12 | Ramsey Canyon, Huachuca Mts, AZ |  |
| *Adineta*sp. | Adi3 | RamO1.11 | Ramsey Canyon, Huachuca Mts, AZ |  |
| *Adineta*sp. | Adi4 | Bird2.3 | Bird bath, Tucson, AZ | DQ078518 |
| *Adineta*sp. | Adi4 | Mem1.8 | Memphis, TN |  |
| *Adineta*sp. | Adi4 | Mem1.9 | Memphis, TN |  |
| *Adineta*sp. | Adi4 | Mem1.10 | Memphis, TN |  |
| *Adineta*sp. | Adi5 | Mem1.1 | Memphis, TN |  |
| *Adineta*sp. | Adi5 | LRatM1.2 | Little Rat Pool, Virginia Dale, CO |  |
| *Adineta*sp. | Adi5 | LRat1.2 | Little Rat Pool, Virginia Dale, CO |  |
| *Adineta vaga* | Adi singlet | WAv1.1 | Milan, Italy^a^ | DQ078512 |
| *Adineta oculata* | Adi singlet | War1.1 | Unknown | DQ078515 |
| *Adineta*sp. | Adi singlet | BCB1.2b | Big Casa Blanca, Santa Rita Mountains, AZ | DQ078519 |
| *Adineta ricciae* | Adi singlet | AdiRic1.1 | Ryan's Billabong, NSA, Australia |  |
| *Habrotrocha* sp. | Hab1 | Glen1.10 | Glenwood, NM | DQ078524 |
| *Habrotrocha* sp. | Hab1 | Glen1.5 | Glenwood, NM | DQ078525 |
| *Habrotrocha* sp. | Hab1 | Pim1.1b | Pima Canyon, Santa Catalina Mts, AZ |  |
| *Habrotrocha* sp. | Hab1 | Rob2.10d2 | Robinson Spring, Santa Rita Mts, AZ |  |
| *Habrotrocha* sp. | Hab2 | Rob2.7 | Robinson Spring, Santa Rita Mts, AZ | DQ078527 |
| *Habrotrocha* sp. | Hab2 | Rob2.8 | Robinson Spring, Santa Rita Mts, AZ | DQ078526 |
| *Habrotrocha* sp. | Hab2 | Rob2.9 | Robinson Spring, Santa Rita Mts, AZ | DQ078528 |
| *Habrotrocha* sp. | Hab2 | HuH1.5b | Hex Pool, Hueco Tanks State Park, TX | DQ078529 |
| *Habrotrocha* sp. | Hab2 | Rob2.10a | Robinson Spring, Santa Rita Mts, AZ | DQ078530 |
| *Habrotrocha* sp. | Hab2 | Rob2.10c | Robinson Spring, Santa Rita Mts, AZ | DQ078532 |
| *Habrotrocha* sp. | Hab3 | Wad1.7 | Wading pool, Tucson, AZ | DQ078533 |
| *Habrotrocha* sp. | Hab3 | Wad1.9 | Wading pool, Tucson, AZ | DQ078534 |
| *Habrotrocha* sp. | Hab4 | Smok1.1 | Seep, Great Smoky Mountain NP, TN | DQ078521 |
| *Habrotrocha* sp. | Hab4 | Smok1.2 | Seep, Great Smoky Mountain NP, TN | DQ078522 |
| *Habrotrocha* sp. | Hab5 | LRatM1.1 | Little Rat Pool, Virginia Dale, CO |  |
| *Habrotrocha* sp. | Hab5 | LRat1.5 | Little Rat Pool, Virginia Dale, CO |  |
| *Habrotrocha* sp. | Hab6 | WagM1.22 | Arboretum, Waginengen, Netherlamds |  |
| *Habrotrocha* sp. | Hab6 | WagM1.23 | Arboretum, Waginengen, Netherlamds |  |
| *Habrotrocha* sp. | Hab6 | WagM1.24 | Arboretum, Waginengen, Netherlamds |  |
| *Habrotrocha* sp. | Hab6 | WagM1.25* | Arboretum, Waginengen, Netherlamds |  |
| *Habrotrocha* sp. | Hab6 | WagM1.26* | Arboretum, Waginengen, Netherlamds |  |
| *Habrotrocha* sp. | Hab6 | WagM1.27* | Arboretum, Waginengen, Netherlamds |  |
| *Habrotrocha* sp. | Hab6 | WagM1.28* | Arboretum, Waginengen, Netherlamds |  |
| *Habrotrocha* sp. | Hab6 | WagM1.29 | Arboretum, Waginengen, Netherlamds |  |
| *Habrotrocha* sp. | Hab7 | SeaR2.1 | Creek, Sea Ranch, California |  |
| *Habrotrocha* sp. | Hab7 | SeaR2.2 | Creek, Sea Ranch, California |  |
| *Habrotrocha constricta* | Hab singlet | WHc1.1 | Sandwich, MA^c^ | DQ078520 |
| *Habrotrocha* sp. | Hab singlet | Pim1.1 | Pima Canyon, Santa Catalina Mts, AZ |  |
| *Habrotrocha* sp. | Hab singlet | Rob2.10d | Robinson Spring, Santa Rita Mts, AZ |  |
| *Habrotrocha* sp. | Hab singlet | Cent1.1 | Central Park, New York, NY |  |
| *Habrotrocha* sp. | Hab singlet | WagM1.15 | Arboretum, Waginengen, Netherlamds |  |
| *Habrotrocha* sp. | Hab singlet | SeaR2.2*** | Creek, Sea Ranch, California |  |
| *Macrotrachela* sp. | Mac1 | HuH1.1 | Hex Pool, Hueco Tanks State Park, TX | DQ078538 |
| *Macrotrachela* sp. | Mac1 | HuJu1.10 | Julie's Pool, Hueco Tanks State Park, TX | DQ078540 |
| *Macrotrachela* sp. | Mac1 | HuJu1.11 | Julie's Pool, Hueco Tanks State Park, TX |  |
| *Macrotrachela* sp. | Mac1 | HuJu1.12 | Julie's Pool, Hueco Tanks State Park, TX | DQ078539 |
| *Macrotrachela* sp. | Mac2 | SnoB1.3 | Snowmelt, Snowy Range, WY | DQ078541 |
| *Macrotrachela* sp. | Mac2 | SnoB1.7 | Snowmelt, Snowy Range, WY | DQ078542 |
| *Macrotrachela* sp. | Mac2 | SnoB1.9 | Snowmelt, Snowy Range, WY |  |
| *Macrotrachela* sp. | Mac3 | MyBa1.6 | Bird bath, Mt. Prospect, IL |  |
| *Macrotrachela* sp. | Mac3 | MyBa1.8 | Bird bath, Mt. Prospect, IL |  |
| *Macrotrachela* sp. | Mac3 | MyBa1.3 | Bird bath, Mt. Prospect, IL |  |
| *Macrotrachela quadricornifera* | Mac singlet | WMq1.1 | Italy^a^ | DQ078536 |
| *Macrotrachela* sp. | Mac singlet | Shmoo1.11 | Shmoo Pool, Virginia Dale, CO |  |
| *Macrotrachela* sp. | Mac singlet | RamO1.6 | Ramsey Canyon, Huachuca Mts, AZ |  |
| *Macrotrachela* sp. | Mac singlet | Rat2.4 | Rat Cave Pool, Virginia Dale, CO |  |
| *Macrotrachela* sp. | Mac singlet | MyBa1.1 | Bird bath, Mt. Prospect, IL |  |
| *Macrotrachela* sp. | Mac singlet | MyBa1.7 | Bird bath, Mt. Prospect, IL |  |
| *Philodina roseola* | Pha1 | WPr1.1 | unknown^d^ | DQ078544 |
| *Philodina roseola* | Pha1 | WPr1.1a | unknown^d^ |  |
| *Philodina roseola* | Pha1 | WPr1.1b | unknown^d^ |  |
| *Philodina* sp. | Pha1 | Car1.1 | unknown^e^ | DQ078545 |
| *Philodina* sp. | Pha1 | Car.Pr2 | unknown^e^ | DQ078546 |
| *Philodina* sp. | Pha2 | Car.Pr1 | unknown^e^ | DQ078547 |
| *Philodina* sp. | Pha2 | Huf1.1 | Pool f, Hueco Tanks State Park, TX | DQ078549 |
| *Philodina* sp. | Pha2 | Huf1.2 | Pool f, Hueco Tanks State Park, TX | DQ078550 |
| *Philodina* sp. | Pha2 | Huf1.3 | Pool f, Hueco Tanks State Park, TX | DQ078551 |
| *Philodina* sp. | Pha2 | HuJ1.1 | Jenn's Pool, Hueco Tanks State Park, TX | DQ078548 |
| *Philodina* sp. | Pha2 | HuJ1.3 | Jenn's Pool, Hueco Tanks State Park, TX |  |
| *Philodina* sp. | Pha3 | Chi1.1 | Chino Canyon, Santa Rita Mts, AZ | DQ078564 |
| *Philodina* sp. | Pha3 | Duc1.1 | Duck pond, Sierra Vista, AZ | DQ078556 |
| *Philodina* sp. | Pha3 | Duc1.2 | Duck pond, Sierra Vista, AZ | DQ078557 |
| *Philodina* sp. | Pha3 | FlT1.1 | Florida Canyon, Santa Rita Mts, AZ |  |
| *Philodina* sp. | Pha3 | FlT2.1 | Florida Canyon, Santa Rita Mts, AZ | DQ078552 |
| *Philodina* sp. | Pha3 | FlT2.2 | Florida Canyon, Santa Rita Mts, AZ | DQ078560 |
| *Philodina* sp. | Pha3 | FlT2.3c | Florida Canyon, Santa Rita Mts, AZ | DQ078561 |
| *Philodina* sp. | Pha3 | FRP1.3 | Unnamed canyon, Santa Catalina Mts, AZ | DQ078559 |
| *Philodina* sp. | Pha3 | Hel1.10 | Helvetia, Santa Rita Mts, AZ |  |
| *Philodina* sp. | Pha3 | Hel1.3 | Helvetia, Santa Rita Mts, AZ | DQ078590 |
| *Philodina* sp. | Pha3 | HuK1.1 | Kettle Pool, Hueco Tanks State Park, TX | DQ078562 |
| *Philodina* sp. | Pha3 | HuK1.2 | Kettle Pool, Hueco Tanks State Park, TX | DQ078563 |
| *Philodina* sp. | Pha3 | HuK1.3b | Kettle Pool, Hueco Tanks State Park, TX |  |
| *Philodina* sp. | Pha3 | HuN1.1 | North Pool, Huech Tanks State Park, TX | DQ078553 |
| *Philodina* sp. | Pha3 | HuN1.2 | North Pool, Huech Tanks State Park, TX | DQ078554 |
| *Philodina* sp. | Pha3 | PaP1.3 | Paton's pond, Patagonia, AZ | DQ078558 |
| *Philodina* sp. | Pha3 | ScM1.1 | Scotia Canyon, Huachucha Mts, AZ | DQ078555 |
| *Philodina* sp. | Pha3 | SevF1.2 | Seven Falls, Catalina Mts, AZ |  |
| *Philodina* sp. | Pha3 | SevF1.3 | Seven Falls, Catalina Mts, AZ |  |
| *Philodina* sp. | Pha3 | SevF1.4 | Seven Falls, Catalina Mts, AZ |  |
| *Philodina* sp. | Pha3 | VenD1.3 | Ventana Canyon dam, Santa Catalina Mts, AZ |  |
| *Philodina* sp. | Pha3 | VenD1.4 | Ventana Canyon dam, Santa Catalina Mts, AZ |  |
| *Philodina* sp. | Pha3 | Wil1.1a | Wilderness of Rocks, Santa Catalina Mts, AZ |  |
| *Philodina* sp. | Pha3 | Wil1.2 | Wilderness of Rocks, Santa Catalina Mts, AZ | DQ078565 |
| *Philodina* sp. | Pha3 | Wil1.3 | Wilderness of Rocks, Santa Catalina Mts, AZ | DQ078566 |
| *Philodina* sp. | Pha4 | Amp1.1 | Pontatoc Canyon, Santa Catalina Mts, AZ | DQ078580 |
| *Philodina* sp. | Pha4 | Amp1.2 | Pontatoc Canyon, Santa Catalina Mts, AZ | DQ078581 |
| *Philodina* sp. | Pha4 | Amp1.3 | Pontatoc Canyon, Santa Catalina Mts, AZ | DQ078582 |
| *Philodina* sp. | Pha4 | Ven1.1 | Ventana Canyon, Santa Catalina Mts, AZ |  |
| *Philodina* sp. | Pha4 | Ven1.2 | Ventana Canyon, Santa Catalina Mts, AZ | DQ078583 |
| *Philodina* sp. | Pha4 | Ven1.3 | Ventana Canyon, Santa Catalina Mts, AZ | DQ078578 |
| *Philodina* sp. | Pha4 | Yet1.1 | Yetman trail, Tucson Mts, AZ | DQ078568 |
| *Philodina* sp. | Pha4 | Yet1.2 | Yetman trail, Tucson Mts, AZ | DQ078579 |
| *Philodina* sp. | Pha4 | Yet1.3 | Yetman trail, Tucson Mts, AZ | DQ078569 |
| *Philodina* sp. | Pha4 | Yet2.1 | Yetman trail, Tucson Mts, AZ | DQ078570 |
| *Philodina* sp. | Pha4 | Yet2.10 | Yetman trail, Tucson Mts, AZ | DQ078577 |
| *Philodina* sp. | Pha4 | Yet2.2 | Yetman trail, Tucson Mts, AZ | DQ078571 |
| *Philodina* sp. | Pha4 | Yet2.3 | Yetman trail, Tucson Mts, AZ | DQ078595 |
| *Philodina* sp. | Pha4 | Yet2.4 | Yetman trail, Tucson Mts, AZ | DQ078572 |
| *Philodina* sp. | Pha4 | Yet2.6 | Yetman trail, Tucson Mts, AZ | DQ078573 |
| *Philodina* sp. | Pha4 | Yet2.7 | Yetman trail, Tucson Mts, AZ | DQ078574 |
| *Philodina* sp. | Pha4 | Yet2.8 | Yetman trail, Tucson Mts, AZ | DQ078575 |
| *Philodina* sp. | Pha4 | Yet2.9 | Yetman trail, Tucson Mts, AZ | DQ078576 |
| *Philodina* sp. | Pha5 | Kof1.1 | Palm Canyon, Kofa NWR, AZ | DQ078586 |
| *Philodina* sp. | Pha5 | Kof1.2 | Palm Canyon, Kofa NWR, AZ | DQ078587 |
| *Philodina* sp. | Pha5 | Kof1.4 | Palm Canyon, Kofa NWR, AZ | DQ078584 |
| *Philodina* sp. | Pha5 | Kof1.5 | Palm Canyon, Kofa NWR, AZ | DQ078585 |
| *Philodina* sp. | Pha6 | Bird2.2 | Tucson, AZ | DQ078592 |
| *Philodina* sp. | Pha6 | BirdE1.1 | Tucson, AZ | DQ078593 |
| *Philodina* sp. | Pha6 | Yet2.5 | Yetman trail, Tucson Mts, AZ | DQ078591 |
| *Philodina* sp. | Pha8 | Gut1.1c | Rain gutter, Columbus, OH | DQ078598 |
| *Philodina* sp. | Pha8 | Gut1b1b | Rain gutter, Columbus, OH | DQ078597 |
| *Philodina* sp. | Pha8 | Hel1.2 | Helvetia, Santa Rita Mts, AZ | DQ078600 |
| *Philodina* sp. | Pha8 | Rou1.3 | Round Valley, Chniricahua Mts, AZ | DQ078599 |
| *Philodina* sp. | Pha8 | Rou1.10 | Round Valley, Chniricahua Mts, AZ | DQ078601 |
| *Philodina* sp. | Pha8 | Shmoo3.18 | Shmoo Pool, Virginia Dale, CO |  |
| *Philodina* sp. | Pha8 | Shmoo3.15 | Shmoo Pool, Virginia Dale, CO |  |
| *Philodina* sp. | Pha8 | Shmoo3.4 | Shmoo Pool, Virginia Dale, CO |  |
| *Philodina* sp. | Pha9 | Bel1.1 | Bellows Spring, Santa Rita Mts, AZ | DQ078606 |
| *Philodina* sp. | Pha9 | Bel1.2 | Bellows Spring, Santa Rita Mts, AZ | DQ078602 |
| *Philodina* sp. | Pha9 | Bel1.4 | Bellows Spring, Santa Rita Mts, AZ | DQ078607 |
| *Philodina* sp. | Pha9 | Bel1.5 | Bellows Spring, Santa Rita Mts, AZ | DQ078603 |
| *Philodina* sp. | Pha9 | Bel1.8 | Bellows Spring, Santa Rita Mts, AZ | DQ078604 |
| *Philodina* sp. | Pha9 | Bel1.10 | Bellows Spring, Santa Rita Mts, AZ | DQ078605 |
| *Philodina* sp. | Pha9 | Bel1.11 | Bellows Spring, Santa Rita Mts, AZ |  |
| *Philodina* sp. | Pha9 | Bel2.1 | Bellows Spring, Santa Rita Mts, AZ | DQ078609 |
| *Philodina* sp. | Pha9 | Bel2.5 | Bellows Spring, Santa Rita Mts, AZ | DQ078610 |
| *Philodina* sp. | Pha9 | Bel2.6 | Bellows Spring, Santa Rita Mts, AZ | DQ078611 |
| *Philodina* sp. | Pha9 | Bel2.8 | Bellows Spring, Santa Rita Mts, AZ | DQ078608 |
| *Philodina* sp. | Pha10 | Angl1.2b | Angelfish Pool, Virginia Dale, CO |  |
| *Philodina* sp. | Pha10 | Angl1.3 | Angelfish Pool, Virginia Dale, CO | DQ078618 |
| *Philodina* sp. | Pha10 | BuP1.2 | Bull Pasture, Organ Pipe NM, AZ |  |
| *Philodina* sp. | Pha10 | Rat1.1 | Rat Cave Pool, Virginia Dale, CO | DQ078615 |
| *Philodina* sp. | Pha10 | Rat1.2 | Rat Cave Pool, Virginia Dale, CO | DQ078616 |
| *Philodina* sp. | Pha10 | Rat1.3 | Rat Cave Pool, Virginia Dale, CO | DQ078617 |
| *Philodina* sp. | Pha10 | Rat2.2 | Rat Cave Pool, Virginia Dale, CO |  |
| *Philodina* sp. | Pha10 | Rou1.2 | Round Valley, Chniricahua Mts, AZ | DQ078613 |
| *Philodina* sp. | Pha10 | Rou1.4 | Round Valley, Chniricahua Mts, AZ | DQ078614 |
| *Philodina* sp. | Pha10 | Rou1.5 | Round Valley, Chniricahua Mts, AZ |  |
| *Philodina* sp. | Pha10 | Rou2.2 | Round Valley, Chniricahua Mts, AZ |  |
| *Philodina* sp. | Pha11 | MedBP1.1 | Medicine Bow Peak, Medicine Bow Mts, WY |  |
| *Philodina* sp. | Pha11 | MedBP1.2 | Medicine Bow Peak, Medicine Bow Mts, WY |  |
| *Philodina* sp. | Pha11 | MedBP1.3 | Medicine Bow Peak, Medicine Bow Mts, WY |  |
| *Philodina* sp. | Pha11 | MedBP1.6 | Medicine Bow Peak, Medicine Bow Mts, WY |  |
| *Philodina* sp. | Pha11 | SnoE1.8 | Snowmelt, Medicine Bow Mts, WY |  |
| *Philodina* sp. | Pha11 | SnoE1.3 | Snowmelt, Medicine Bow Mts, WY |  |
| *Philodina* sp. | Pha12 | Bear1.2 | Bear Canyon, Santa Catalina Mts, AZ | DQ078619 |
| *Philodina* sp. | Pha12 | Rat2.3 | Rat Cave Pool, Virginia Dale, CO |  |
| *Philodina* sp. | Pha13 | Kof1.3 | Palm Canyon, Kofa NWR, AZ | DQ078588 |
| *Philodina* sp. | Pha13 | Kof1.6 | Palm Canyon, Kofa NWR, AZ | DQ078589 |
| *Philodina* sp. | Pha14 | BirdT1.3 | Bird bath, Tucson, AZ | DQ078594 |
| *Philodina* sp. | Pha14 | BirdT1.6 | Bird bath, Tucson, AZ |  |
| *Philodina* sp. | Pha15 | Aqua1.1 | Aquarium, Superior, AZ |  |
| *Philodina* sp. | Pha15 | Aqua1.2 | Aquarium, Superior, AZ |  |
| *Philodina* sp. | Pha16 | Wai1.1 | Waimea, HA |  |
| *Philodina* sp. | Pha16 | Wai1.2 | Waimea, HA |  |
| *Philodina* sp. | Pha17 | Angl2.1 | Angelfish Pool, Virginia Dale, CO |  |
| *Philodina* sp. | Pha17 | LRatM1.3 | Little Rat Cave Pool, Virginia Dale, CO |  |
| *Philodina* sp. | Pha17 | Angl2.2 | Angelfish Pool, Virginia Dale, CO |  |
| *Philodina* sp. | Pha17 | Angl3.2 | Angelfish Pool, Virginia Dale, CO |  |
| *Philodina* sp. | Pha17 | Angl3.12 or 15 | Angelfish Pool, Virginia Dale, CO |  |
| *Philodina* sp. | Pha17 | Angl3.3 | Angelfish Pool, Virginia Dale, CO |  |
| *Philodina* sp. | Pha17 | Angl3.12 | Angelfish Pool, Virginia Dale, CO |  |
| *Philodina* sp. | Pha17 | Angl3.8 | Angelfish Pool, Virginia Dale, CO |  |
| *Philodina* sp. | Pha17 | Angl3.14 | Angelfish Pool, Virginia Dale, CO |  |
| *Philodina* sp. | Pha singlet | BCG1.2g | Big Casa Blanca, Santa Rita Mts, AZ |  |
| *Philodina* sp. | Pha singlet | Bear1.1 | Bear Canyon, Santa Catalina Mts, AZ | DQ078620 |
| *Philodina* sp. | Pha singlet | Bird1.1 | Bird bath, Tucson, AZ | DQ078596 |
| *Philodina* sp. | Pha singlet | Fin1.1 | Finger Rock Canyon, Santa Catalina Mts, AZ | DQ078612 |
| *Philodina* sp. | Pha singlet | FlT3.3 | Florida Canyon, Santa Rita Mts, AZ | DQ078621 |
| *Philodina* sp. | Pha singlet | SwT1.2 | Sweetwater Wetlands, Tucson, AZ | DQ078567 |
| *Philodina* sp. | Pha singlet | Trap1.2 | Trap Lake, Front Range, CO | DQ078537 |
| *Philodina* sp. | Pha singlet | Wai.1.3a | Waimea, HA |  |
| *Rotaria* sp. nov. | Rot1 | SnoE1.4 | Snowy Range, WY |  |
| *Rotaria* sp. nov. | Rot1 | SnoE1.5 | Snowy Range, WY |  |
| *Rotaria* sp. nov. | Rot2 | Ari1.2 | Arivaca Lake, AZ |  |
| *Rotaria* sp. nov. | Rot2 | Ari1.3 | Arivaca Lake, AZ |  |
| *Rotaria* sp. nov. | Rot3 | UIL1.4 | University of Illinois arboretum pond, IL |  |
| *Rotaria* sp. nov. | Rot3 | UIL1.2 | University of Illinois arboretum pond, IL |  |
| *Rotaria* sp. nov. | Rot4 | Mem1.5 | Memphis, TN |  |
| *Rotaria* sp. nov. | Rot4 | Mem1.7 | Memphis, TN |  |
| *Rotaria* sp. nov. | Rot5 | WagM1.12 | Arboretum, Waginengen, Netherlamds |  |
| *Scepanotrocha* sp. | Rot5 | WagM1.13 | Arboretum, Waginengen, Netherlamds |  |
| Unidentified | Rot singlets | Cre1.2 | Creek, Chicago, IL |  |
| Unidentified | Rot singlets | Trap1.1 | Trap Lake, Front Range, CO | DQ078543 |
| Unidentified | Sce singlet | Shmoo 2.6 | Shmoo Pool, Virginia Dale, CO |  |
| Unidentified | McBS clade | McBS1.2 | McBeth Spring, Santa Rita Mts, AZ |  |
| Unidentified | McBS clade | McBS1.3 | McBeth Spring, Santa Rita Mts, AZ |  |
| Unidentified | SnoF clade | SnoF1.1 | Libby Flats, Snowy Range, WY |  |
| Unidentified | SnoF clade | SnoF1.3 | Libby Flats, Snowy Range, WY |  |
| Unidentified | singlet | McBM1.11 | McBeth Spring, Santa Rita Mts, AZ |  |
| Unidentified | singlet | WagM1/6 | Arboretum, Waginengen, Netherlamds |  |
| Unidentified | singlet | RamM1/3 | Ramsey Canyon, Huachuca Mts, AZ |  |
| Unidentified | singlet | MyBa2/1 | Bird bath, Mt. Prospect, IL |  |
|  |  |  |  |  |
| ^a^ From Claudia Ricci and Giulio Melone via David Mark Welch | | |  |  |
| ^b^ Purchased from Ward Natural Science Establishment | | |  |  |
| ^c^ From David Mark Welch |  |  |  |  |
| ^d^ From Carolina Biological Supply Company via David Mark Welch | | | |  |
| ^e^ Purchased from Carolina Biological Supply Company | | |  |  |
