# Supplementary material for: Using Population Genetic Theory and DNA Sequences for Species Detection and Identification in Asexual Organisms
Source: PLoS One. 2010 May 13;5(5):e10609. doi: 10.1371/journal.pone.0010609 (PMC2869354; doi:10.1371/journal.pone.0010609)
Supplement: Table S2 — Fungus Penicillium. Some examples of evolutionary species identified as described in the text. (0.03 MB DOC) [file pone.0010609.s006.doc]

Table S2. Fungus *Penicillium*. Some examples of evolutionary species identified as described in the text.

| Named species | π              θ  | K | P |  |
| --- | --- | --- | --- | --- |
| *P. bialowiezense* | 0.0000874 0.0000874 | 0.02752 | > 0.99 |  |
| *P. brevicompactum* | 0.0001223 0.0001223 | 0.02569 | > 0.99 |  |
| *P. olsonii* | 0.000915 0.0009139 | 0.02569 | > 0.99 |  |
| *P. atrimentosum* | 0.0067256 0.0066652 | 0.04771 | > 0.99 |  |
| P. bialowiezense, P. brevicompactum, and P. olsonii are sister clades and K is the distance from named species to the closest member of the clade.For P. atrimentosum, K is the shortest distance to a member of another clade, which in this case is P. glandicola. | | | |  |
|  | | | | |
