# Supplementary material for: Using Population Genetic Theory and DNA Sequences for Species Detection and Identification in Asexual Organisms
Source: PLoS One. 2010 May 13;5(5):e10609. doi: 10.1371/journal.pone.0010609 (PMC2869354; doi:10.1371/journal.pone.0010609)
Supplement: Table S3 — Heterotrophic flagellates. Four named species each consist of two clades that can be assigned to different evolutionary species with high probability. (0.02 MB DOC) [file pone.0010609.s007.doc]

Table S3. Heterotrophic flagellates. Four named species each consist of two clades that can be assigned to different evolutionary species with high probability.

|  | Clade 1 | Clade 2 |  |  | | |
| --- | --- | --- | --- | --- | --- | --- |
| Named species | π              θ  | π              θ  | K | | P | |
| *Rhynchomonas nasuta* | 0.0160 0.0163 | singlet | 0.0644 | | 0.95 > 0.94 | |
| *Ancyromonas sigmoides* | 0.0154 0.0157 | 0.0064 0.0258 | 0.0645 | | > 0.99 | |
| *Caecitellus parvulus* | 0.0012 0.0012 | 0.0012 0.0012 | 0.1276 | | > 0.99 | |
| *Cafeteria* spp. | 0.0014 0.0014 | singlet | 0.221 | | > 0.99 | |
| *R. nasuta* Clade 1 =AF174377, AF174378, HFCC3.3, HFCC18, HFCC302; Singlet HFCC99 | | | | | | |
| *A. sigmoides* Clade 1 = HFCC60, HFCC63, HFCC104; Clade 2 = AF053088, AF174363 | | | | | | |
| *C. parvulus* Clade 1 = AF174367, AF174368; Clade 2 = HFCC300, HFCC301 | | | | | |  |
| *Cafeteria* spp. Clade 1 = AF174364, AF174365, HFCC32, HFCC33, HFCC34, L27633; Singlet AF174366 | | | | | | |
