# Supplementary material for: Using Population Genetic Theory and DNA Sequences for Species Detection and Identification in Asexual Organisms
Source: PLoS One. 2010 May 13;5(5):e10609. doi: 10.1371/journal.pone.0010609 (PMC2869354; doi:10.1371/journal.pone.0010609)
Supplement: Table S4 — Ostreococcus tauri. Clades A and D, and clades B and C, are pairs of sister clades that can be assigned to different evolutionary species with high probability. (0.02 MB DOC) [file pone.0010609.s008.doc]

Table S4. *Ostreococcus tauri*. Clades A and D, and clades B and C, are pairs of sister clades that can be assigned to different evolutionary species with high probability.

|  | Clade 1 | | Clade 2 |  |  |
| --- | --- | --- | --- | --- | --- |
| Clade 1, Clade 2 | π            θ  | π            θ  | | K | P |
| A, D | 0.0165 0.0168 | 0.0250 0.0259 | | 0.6194 | > 0.99 |
| B, C | 0.0176 0.0180 | Singlet | | 0.3394 | > 0.99 |

Clades A-C are those identified by Rodriguez et al. 2005
